# Fetal Myocardium in the Kidney Capsule: An In Vivo Model of Repopulation of Myocytes by Bone Marrow Cells

**DOI:** 10.1371/journal.pone.0031099

**Published:** 2012-02-23

**Authors:** Eric Y. Zhang, Qiang Xiong, Lei Ye, Piradeep Suntharalingam, Xiaohong Wang, C. Michael Astle, Jianyi Zhang, David E. Harrison

**Affiliations:** 1 Division of Cardiology, Department of Medicine, University of Minnesota Medical School, Minneapolis, Minnesota, United States of America; 2 The Jackson Laboratory, Bar Harbor, Maine, United States of America; Northwestern University, United States of America

## Abstract

Debate surrounds the question of whether the heart is a post-mitotic organ in part due to the lack of an *in vivo* model in which myocytes are able to actively regenerate. The current study describes the first such mouse model — a fetal myocardial environment grafted into the adult kidney capsule. Here it is used to test whether cells descended from bone marrow can regenerate cardiac myocytes. One week after receiving the fetal heart grafts, recipients were lethally irradiated and transplanted with marrow from green fluorescent protein (GFP)-expressing C57Bl/6J (B6) donors using normal B6 recipients and fetal donors. Levels of myocyte regeneration from GFP marrow within both fetal myocardium and adult hearts of recipients were evaluated histologically. Fetal myocardium transplants had rich neovascularization and beat regularly after 2 weeks, continuing at checkpoints of 1, 2, 4, 6, 8 and12 months after transplantation. At each time point, GFP-expressing rod-shaped myocytes were found in the fetal myocardium, but only a few were found in the adult hearts. The average count of repopulated myocardium with green rod-shaped myocytes was 996.8 cells per gram of fetal myocardial tissue, and 28.7 cells per adult heart tissue, representing a thirty-five fold increase in fetal myocardium compared to the adult heart at 12 months (when numbers of green rod-shaped myocytes were normalized to per gram of myocardial tissue). Thus, bone marrow cells can differentiate to myocytes in the fetal myocardial environment. The novel *in vivo* model of fetal myocardium in the kidney capsule appears to be valuable for testing repopulating abilities of potential cardiac progenitors.

## Introduction

For many years, the common textbook beliefs have been that no new cardiac myocytes are generated after birth in mammals; that cardiac myocytes are terminally differentiated, beat continuously and rarely fatigue or die; and that the heart increases in size only through hypertrophy. These beliefs were supported by the absence of mitotic figures in myocytes as well as the absence of new cardiac myocytes after cell loss caused by infarction. Recent studies, however, have challenged these beliefs, suggesting that cardiac myocytes are replaced throughout the lifespan [1]–[5], that myocytes can regenerate from resident cardiac progenitor cells (CPCs) [6] as well as from bone marrow [3], [7]–[12], and that the human heart contains cycling myocytes undergoing mitosis and cytokinesis under normal and pathological conditions [1], [2], [13]. Nevertheless, the importance of myocardial regeneration to cardiac health remains controversial, and the absence of an *in vivo* model in which myocytes are actively regenerating has been a significant obstacle in characterizing cardiac progenitor cells.

During normal development, CPCs actively proliferate and differentiate into cardiac myocytes in the fetal myocardium. In the present study, we created an *in vivo* model — fetal myocardium within the kidney capsule — which beat continuously for 12 months. We applied this model to examine the hypothesis that bone marrow with green fluorescence protein (GFP) expression could regenerate cardiomyocytes, thus producing myocytes. As predicted, significant numbers of GFP-expressing rod-shaped myocytes were observed in the fetal myocardium model from 2 weeks to 12 months after irradiation and GFP-labeled marrow transplantation.

## Materials and Methods

### Animals

Animal study protocols were approved by the IACUCs at the University of Minnesota and The Jackson Laboratory (Bar Harbor, ME). Mice were produced, and surgery, irradiation and marrow transplantation were performed in the D1 barrier colony at The Jackson Laboratory (Bar Harbor, ME). Mice were fed an irradiated formulation of the NIH-31 (4% fat) diet, Purina LabDiet's 5LG6 (TestDiet inc. Richmond, IN). All mice were on the C57Bl/6J (B6) (JAX® Mice stock # 000664) inbred strain background. GFP-marked marrow donors were C57BL/6-Tg(UBC-GFP)30Scha/J (JAX® Mice stock # 004353).

### Fetal heart transplantation

B6 mice at 10–12 weeks of age, ranging from 30–40 g in weight, were given 0.60–0.85 ml tribromoethanol anesthetic diluted in sterile phosphate buffered saline by intraperitoneal injection (400 mg/kg, ip). The fur was then clipped from an area about 5 cm^2^, providing a surgical field that was disinfected with 70% ethanol and betadine. Rimadyl (Carprofen, Pfizer) was diluted to a working dilution of 0.5 mg/ml with sterile water and given subcutaneously (5 mg/kg). The mouse was then placed in the right lateral recumbent position. A 5–8 mm incision in the skin was made below the lowest rib, exposing the abdominal wall; a 4–7 mm incision was then made in the abdominal wall. The kidney was exposed and elevated. A small cut was made in the capsule over the lateral border of the kidney with a #5 Dumont forceps and enlarged as the fetal heart (E12–E16 from same background) was inserted deeply into the capsule. The kidney was then replaced in its normal position and the incisions in skin and abdominal wall were closed with 5-0 absorbable suture. Mice were then given 0.5 ml sterile PBS ip and placed under a warming lamp until they responded and moved normally.

### Lethal irradiation and bone marrow transplantation

Seven days after fetal hearts were transplanted, mice were lethally irradiated (1100 rads at a rate of 100 rad/min) using a Cs-137 Shephard (J. L. Shephard & Associates, Glendale, CA) model irradiator, to remove normal precursor cells so they could be replaced by descendants of bone marrow cells marked with GFP.

Donor bone marrow was taken from femur and tibia, filtered and used as single cell suspensions as described previously [14]–[16]. Recipients were warmed to dilate tail veins. GFP-marked marrow cells were injected iv into the lateral tail veins with a 30 gauge needle, giving each recipient 20 million cells. Previously in our lab, this method of bone marrow transplantation has successfully resulted in long-term repopulation of circulating cells derived from GFP donors [17].

### Immunohistochemistry and evaluation of cardiac repopulation from bone marrow

Recipients that had been lethally irradiated and transplanted with GFP^+^ bone marrow were used for the evaluation of cardiac repopulation from bone marrow. Green rod-shaped myocytes were enumerated at 2 weeks (n = 7) and at 2, 4, 6, 12 months (n = 4 each) after irradiation and the marrow transplantation. Positive and negative controls were compared using additional mice of the same strain, sex and age. Both transplanted fetal hearts and adult hearts were examined for GFP-marked cells. Each adult heart was divided into ∼800 sections as previously described in detail [9], [18], [19], whereas kidney regions containing the fetal hearts were divided into ∼500 sections using a cryostat.

Detailed histology methods employed in this study have been described previously [18], [20], [21]. Briefly, tissues were either cryo-stained or paraffin-stained to characterize the fetal heart transplant and to evaluate the cardiac repopulation. For cryo-staining, kidney tissues with fetal heart transplants were embedded in Tissue-Tek OCT compound (Sakura Finetek, Zoeterwoude, The Netherlands) and then sectioned by cryostat (10 µm in thickness). Endothelial marker von Willebrand factor (vWF) and cardiac marker Troponin T (TnT) were detected using anti-vWF (abcam, 1∶50 dilution) and anti-TnT antibodies (Thermo Scientific, 1∶50 dilution) followed by visualization with species matched secondary antibodies conjugated with either TRITC or FITC (Jackson ImmunoResearch, 1∶100 dilution). ProlongGold+DAPI (Invitrogen) was utilized for slide mounting and nuclei visualization. For paraffin-staining, samples were fixed in 10% formalin followed by paraffin embedding and sectioning at a 5-µm thickness. Sections were subject to antigen retrieval using citrate buffer followed by incubation with antibodies against GFP (Abcam, 1∶20 dilution) and α-sarcomeric actin (α-SA, Sigma Aldrich, 1∶100 dilution). Visualization of staining was achieved by incubation with species-matched secondary antibodies conjugated with either TRITC or FITC (Jackson ImmunoResearch, 1∶100 dilution). Rod-shaped large GFP positive cells co-stained positive with α-SA were defined as differentiated cardiomyocytes from bone marrow. Total numbers of GFP^+^/α-SA^+^ cells were counted, and data are presented as total cells/gram myocardium. One per every sequential ten slides was evaluated for each heart. The total count of GFP^+^ rod shaped myocytes of each organ was obtained through multiplication by 10. All negative findings of detecting the GFP^+^ cells had positive controls using the tissue/organ from eGFP mice. All positive findings of eGFP^+^ cells had their respective own control of eGFP negative cells on the same slide.

### Statistics and data analysis

Levels of myocyte regeneration from bone marrow are represented by the number of green rod-shaped myocytes divided by grams of myocardium, assuming that the size of the fetal heart at E17 is 10% that of an adult heart. All values are expressed as mean ± standard deviation. All statistical analyses were performed in Sigmastat version 3.5 (San Jose, CA). Rod-shaped myocyte data were compared between the fetal and adult hearts using Student's t-test, using the significance level of type I error (P<0.05).

## Results

Both fetal and adult hearts of recipients were evaluated at time points ranging from 2 weeks to 12 months after irradiation and marrow transplantation, as detailed above. Transplanted fetal hearts beat regularly in the kidney capsule at ∼70 beats per minute at time points of 2 weeks (n = 7) and 1, 2, 4, 6, 8 and 12 months (n = 4, at each monthly time point) after the transplantation (Video S1). In addition, we observed vigorous neovascularization of the fetal myocardium under the kidney capsule. At week 1, a mass of blood vessels was localized near the fetal heart (Figure S1) that was a result of angiogenesis of sprouting from the preexisting vessels as they were GFP staining negative. At 2–12 months post fetal transplantation, some of these vessels formed small arteries with visible established circulation. No masses of blood vessels appeared elsewhere on the kidney.

**Figure 1:**
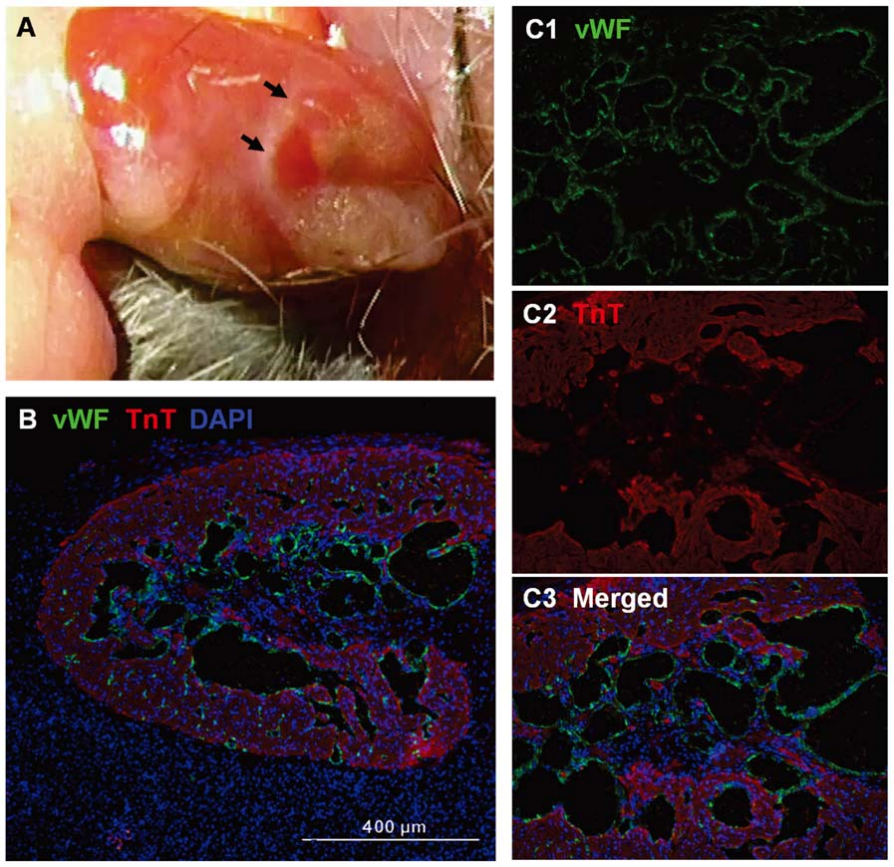
Characterization of fetal heart transplant. A: Typical fetal heart (arrows) on the surface of a recipient kidney 10 months after transplantation into the kidney capsule. B: Cross section of this fetal heart co-stained with vWF (green), TnT (red) and DAPI (blue). C1 and C2: Higher magnification of fetal heart visualized by vWF and TnT staining. C3: Myocardial structure changes with rich channels from the LV cavity that penetrate everywhere across the LV wall.

From 2 through 12 months after transplantation, the explants' histological characteristics are similar; these are illustrated in the first two figures. In Figure 1, Panel A shows a typical fetal heart (arrows) on the surface of a recipient kidney 10 months after transplantation. Panel B illustrates a cross section of this fetal heart co-stained with vWF (green), TnT (red) and DAPI (blue). Panel C shows a higher magnification of the fetal heart. The endocardial cell lining is visualized by vWF staining (Panel C1). It is interesting to note that the myocardial structure changes with rich channels from the LV cavity which penetrate everywhere across the LV wall (Panel C3).

**Figure 2:**
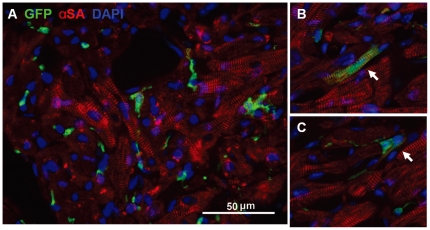
Myocytes transdifferentiation from GFP^+^ bone marrow in fetal heart inside kidney capsule. Immunostaining was performed at 12 months after fetal heart transplantation into kidney capsule and GFP^+^ bone marrow transplantation. A: GFP^+^ circulating cells homed into the fetal heart, which was beating in the kidney capsule. B and C: Two cardiomyocytes (arrows) transdifferentiated from the GFP^+^ bone marrow-origin cells. Striation structure and co-localization of GFP (green) and α-sarcomeric actin (red) are evident.

**Figure 3:**
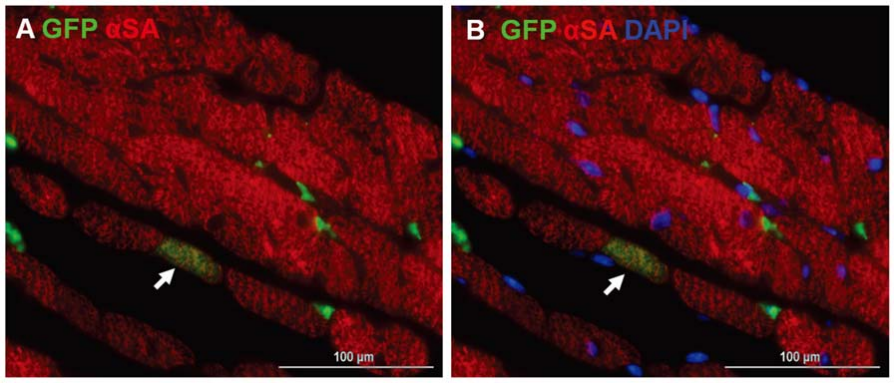
Myocyte transdifferentiation from GFP^+^ bone marrow in adult heart 12 months after GFP^+^ bone marrow transplantation. Co-staining of GFP and α-sarcomeric actin reveals a cardiomyocyte also positive for GFP, suggesting that GFP^+^ bone marrow cells differentiate into cardiomyocyte in adult heart.

**Figure 4:**
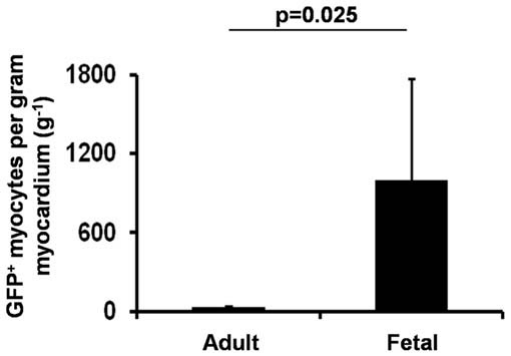
Counts of myocyte transdifferentiation from GFP^+^ bone marrow in adult heart vs. fetal tissue 12 months after fetal heart and GFP^+^ bone marrow transplantation. The proportions of bone marrow descended myocytes in transplanted fetal tissue are 34.7- fold greater than in adult hearts.

Figure 2 illustrates typical staining found in fetal myocardium in the kidney capsule 12 months after transplantation; it is characterized with small mononucleated myocytes with irregular fiber orientation (Figure 2A). Clear GFP^+^ production in myocytes with striation indicates descent from the donor bone marrow (Figure 2 B–C). The bright green rod-shaped large myocytes with distinct size and shape in Figure 2 B–C exclude the possibility that these could be green fluorescent macrophages engulfing recipient fetal origin myocytes. Myocyte differentiation from donor marrow does occur in the adult heart, as shown in Figure 3. In the normal adult heart, however, the very small proportion of green myocytes indicates that bone marrow differentiation to myocytes is a far less frequent event than in the fetal heart. To compare these frequencies at 12 months, numbers of green rod-shaped myocytes found in fetal hearts were compared to adult recipient hearts. It is important to normalize the number of green rod-shaped myocytes per gram of myocardium tissue mass, as adult hearts are at least 10 times larger than fetal hearts. At 12-months of age, the heart weight was 133.8±24.2 mg and 12.6±0.7 mg for the adult- and fetal- heart, respectively. The proportion of bone marrow descended myocytes in transplanted fetal tissue are 34.7- fold greater than in adult hearts (996.8±774.6 vs. 28.7±13.4 per gram of myocardium; Figure 4, P<0.05). This frequency would exclude the cell fusion confounding factor, as it was reported that labeled stem cells fused with myocytes occur at a much lower frequency [22]. However, the data from present study cannot definitively exclude the possibility of mobilized BM cell fusion with the recipient cardiomyocyte, which warrant future investigations.

## Discussion

Increases in cardiac mass in mammals during fetal life occur mainly due to cardiomyocyte proliferation. After birth, the heart has been viewed as a post-mitotic organ consisting of a predetermined number of parenchymal cells that are preserved throughout life [23]–[25]. Adaptive increases in cardiac mass in adults as a result of hemodynamic burden are achieved mainly through increase in cell size known as hypertrophy [23]–[25], as shown by the absence of mitotic figures in myocytes, plus the observation that regions of transmural infarction evolve into essentially avascular, thin collagenous scar tissue. These facts make the suggestion that cardiomyocytes regenerate in adults highly controversial.

Nevertheless, recent studies have challenged the paradigm that heart tissue is incapable of regeneration. Adult human hearts contain myocytes undergoing mitosis and cytokinesis under normal and pathological conditions [13], [26]–[29]. The heart is one of many organ systems that constantly renews and whose capacity to replace cells depends on the persistence of a stem cell compartment [29]–[31]. Such cardiac progenitor cells (CPCs) are responsible for the constant turnover of cardiomyocytes, endothelial cells, smooth muscle cells and fibroblasts. If CPCs are active in adults, however, why is ischemic myocardial damage not spontaneously repaired and heart failure cured? Perhaps CPCs, or cells essential for their support and differentiation, or both, are especially vulnerable. Thus in the infarcted region, they die, preventing regrowth and repair. This would explain why CPC growth only occurs in viable myocardium [26], [32]. In addition, cellular senescence may increase vulnerability to damage and limit repair in older people [33]. Thus, a defective CPC compartment may prevent cardiac repair whether the initiating event is ischemic myocardial injury, aging or diabetes [34].

There are reports that cardiac myocytes can be derived from marrow [35], specifically side population precursor cells following LAD ligation [11], [36]. Transplantation of GFP^+^ lineage negative, c-kit^+^ cells (presumably containing both hematopoietic and mesenchymal stem cell populations) into the ventricular wall after LAD ligation improved function of the ventricle and produced GFP cells with cardiac phenotype in the myocardium [3]. Contradicting these findings, other laboratories using genetic markers have reported that lineage negative, c-kit^+^ marrow cells did not differentiate into cardiomyocytes [22], [37]. Perhaps this contradiction is partly resolved by our demonstration here that bone marrow cells can differentiate to myocytes in the fetal heart environment, but that it is a much rarer event in the adult heart.

Survival of the fetal myocardium in our studies is demonstrated unequivocally by the regular beating (Video S1). However, even the degree of repopulation by marrow in our fetal heart model may represent a minimum. The unusual myocardial structural changes with rich channels rising from the LV cavity and across the LV wall (Figure 1C) in our model may affect myocardial perfusion, reducing myocyte regeneration by marrow-derived precursors. Furthermore, while the B6 recipient is nearly 100% repopulated by hematopoietic donor cells after irradiation and marrow grafts [14]–[16], [38], recipient CPCs may resist irradiation, and their competition may further reduce myocyte regeneration by marrow-derived precursors. While the rod-shaped GFP-marked myocytes in the fetal myocardial tissue demonstrate unequivocally that marrow can produce myocytes (Figure 2), further development of our model may improve cardiac repopulation by injected cells.

In summary, we here demonstrate myocyte regeneration from marrow cells using a novel *in vivo* model of fetal myocardium transplantation within the kidney capsule followed by lethal irradiation plus marrow transplantation from GFP donor mice. The level of regeneration of GFP-marked rod-shaped myocytes was 35 fold greater in fetal myocardium than in the adult heart. This novel *in vivo* model will be valuable for testing repopulating abilities of many different potential cardiac progenitors and for identifying which of multiple cardiac progenitor cell types can produce myocytes most effectively. Our model also will be useful for distinguishing relative myocyte-producing abilities in competitive repopulation comparisons [14]–[16], [38].

## Supporting Information

Video S1 Fetal heart beating in the kidney capsule (10 months after the transplantation, separate video file downloadable from online).
(WMV)

Figure S1 **A mass of blood vessels (highlighted area) was observed near the fetal heart at one week post fetal heart transplantation to the kidney capsule.**
(TIF)

## References

[1] Kajstura J, Urbanek K, Perl S, Hosoda T, Zheng H (2010). Cardiomyogenesis in the adult human heart.. Circ Res.

[2] Leri A, Kajstura J, Anversa P (2011). Role of cardiac stem cells in cardiac pathophysiology: a paradigm shift in human myocardial biology.. Circ Res.

[3] Orlic D, Kajstura J, Chimenti S, Jakoniuk I, Anderson SM (2001). Bone marrow cells regenerate infarcted myocardium.. Nature.

[4] Bergmann O, Bhardwaj RD, Bernard S, Zdunek S, Barnabe-Heider F (2009). Evidence for cardiomyocyte renewal in humans.. Science.

[5] Loffredo FS, Steinhauser ML, Gannon J, Lee RT (2011). Bone marrow-derived cell therapy stimulates endogenous cardiomyocyte progenitors and promotes cardiac repair.. Cell Stem Cell.

[6] Bearzi C, Rota M, Hosoda T, Tillmanns J, Nascimbene A (2007). Human cardiac stem cells.. Proc Natl Acad Sci U S A.

[7] Rota M, Kajstura J, Hosoda T, Bearzi C, Vitale S (2007). Bone marrow cells adopt the cardiomyogenic fate in vivo.. Proc Natl Acad Sci U S A.

[8] Liu J, Hu Q, Wang Z, Xu C, Wang X (2004). Autologous stem cell transplantation for myocardial repair.. Am J Physiol Heart Circ Physiol.

[9] Wang X, Hu Q, Nakamura Y, Lee J, Zhang G (2006). The role of the sca-1+/CD31− cardiac progenitor cell population in postinfarction left ventricular remodeling.. Stem Cells.

[10] Zeng L, Hu Q, Wang X, Mansoor A, Lee J (2007). Bioenergetic and functional consequences of bone marrow-derived multipotent progenitor cell transplantation in hearts with postinfarction left ventricular remodeling.. Circulation.

[11] Pfister O, Mouquet F, Jain M, Summer R, Helmes M (2005). CD31− but Not CD31+ cardiac side population cells exhibit functional cardiomyogenic differentiation.. Circ Res.

[12] Hatzistergos KE, Quevedo H, Oskouei BN, Hu Q, Feigenbaum GS (2010). Bone marrow mesenchymal stem cells stimulate cardiac stem cell proliferation and differentiation.. Circ Res.

[13] Quaini F, Urbanek K, Beltrami AP, Finato N, Beltrami CA (2002). Chimerism of the transplanted heart.. N Engl J Med.

[14] Ertl RP, Chen J, Astle CM, Duffy TM, Harrison DE (2008). Effects of dietary restriction on hematopoietic stem-cell aging are genetically regulated.. Blood.

[15] Harrison DE, Jordan CT, Zhong RK, Astle CM (1993). Primitive hemopoietic stem cells: direct assay of most productive populations by competitive repopulation with simple binomial, correlation and covariance calculations.. Exp Hematol.

[16] Yuan R, Astle CM, Chen J, Harrison DE (2005). Genetic regulation of hematopoietic stem cell exhaustion during development and growth.. Exp Hematol.

[17] Harrison DE, Astle CM (1991). Lymphoid and erythroid repopulation in B6 W-anemic mice: a new unirradiated recipient.. Exp Hematol.

[18] Nakamura Y, Wang X, Xu C, Asakura A, Yoshiyama M (2006). Xenotransplantation of Long Term Cultured Swine Bone Marrow-Derived Mesenchymal Stem Cells.. Stem Cells.

[19] Zhang G, Hu Q, Braunlin EA, Suggs LJ, Zhang J (2008). Enhancing efficacy of stem cell transplantation to the heart with a PEGylated fibrin biomatrix.. Tissue Eng Part A.

[20] Rota M, Padin-Iruegas ME, Misao Y, De Angelis A, Maestroni S (2008). Local activation or implantation of cardiac progenitor cells rescues scarred infarcted myocardium improving cardiac function.. Circ Res.

[21] Xiong Q, Hill KL, Li Q, Suntharalingam P, Mansoor A (2011). A fibrin patch-based enhanced delivery of human embryonic stem cell-derived vascular cell transplantation in a porcine model of postinfarction left ventricular remodeling.. Stem Cells.

[22] Murry CE, Soonpaa MH, Reinecke H, Nakajima H, Nakajima HO (2004). Haematopoietic stem cells do not transdifferentiate into cardiac myocytes in myocardial infarcts.. Nature.

[23] MacLellan WR, Schneider MD (2000). Genetic dissection of cardiac growth control pathways.. Annu Rev Physiol.

[24] Rubart M, Field LJ (2006). Cardiac regeneration: repopulating the heart.. Annu Rev Physiol.

[25] Soonpaa MH, Field LJ (1998). Survey of studies examining mammalian cardiomyocyte DNA synthesis.. Circ Res.

[26] Beltrami AP, Urbanek K, Kajstura J, Yan SM, Finato N (2001). Evidence that human cardiac myocytes divide after myocardial infarction.. N Engl J Med.

[27] Anversa P, Kajstura J (1998). Ventricular myocytes are not terminally differentiated in the adult mammalian heart.. Circ Res.

[28] Nadal-Ginard B, Kajstura J, Leri A, Anversa P (2003). Myocyte death, growth, and regeneration in cardiac hypertrophy and failure.. Circ Res.

[29] Hosoda T, Zheng H, Cabral-da-Silva M, Sanada F, Ide-Iwata N (2011). Human cardiac stem cell differentiation is regulated by a mircrine mechanism.. Circulation.

[30] Anversa P, Sussman MA, Bolli R (2004). Molecular genetic advances in cardiovascular medicine: focus on the myocyte.. Circulation.

[31] Sussman MA, Anversa P (2004). Myocardial aging and senescence: where have the stem cells gone?. Annu Rev Physiol.

[32] Urbanek K, Torella D, Sheikh F, De Angelis A, Nurzynska D (2005). Myocardial regeneration by activation of multipotent cardiac stem cells in ischemic heart failure.. Proc Natl Acad Sci U S A.

[33] Chimenti C, Kajstura J, Torella D, Urbanek K, Heleniak H (2003). Senescence and death of primitive cells and myocytes lead to premature cardiac aging and heart failure.. Circ Res.

[34] Kajstura J, Urbanek K, Rota M, Bearzi C, Hosoda T (2008). Cardiac stem cells and myocardial disease.. J Mol Cell Cardiol.

[35] Bittner RE, Schofer C, Weipoltshammer K, Ivanova S, Streubel B (1999). Recruitment of bone-marrow-derived cells by skeletal and cardiac muscle in adult dystrophic mdx mice.. Anat Embryol (Berl).

[36] Jackson KA, Majka SM, Wang H, Pocius J, Hartley CJ (2001). Regeneration of ischemic cardiac muscle and vascular endothelium by adult stem cells.. J Clin Invest.

[37] Balsam LB, Wagers AJ, Christensen JL, Kofidis T, Weissman IL (2004). Haematopoietic stem cells adopt mature haematopoietic fates in ischaemic myocardium.. Nature.

[38] Chen J, Astle CM, Harrison DE (2000). Genetic regulation of primitive hematopoietic stem cell senescence.. Exp Hematol.

