# Supplementary figures and images for: Fetal Myocardium in the Kidney Capsule: An In Vivo Model of Repopulation of Myocytes by Bone Marrow Cells
Source: PLoS One. 2012 Feb 23;7(2):e31099. doi: 10.1371/journal.pone.0031099 (PMC3285614; doi:10.1371/journal.pone.0031099)

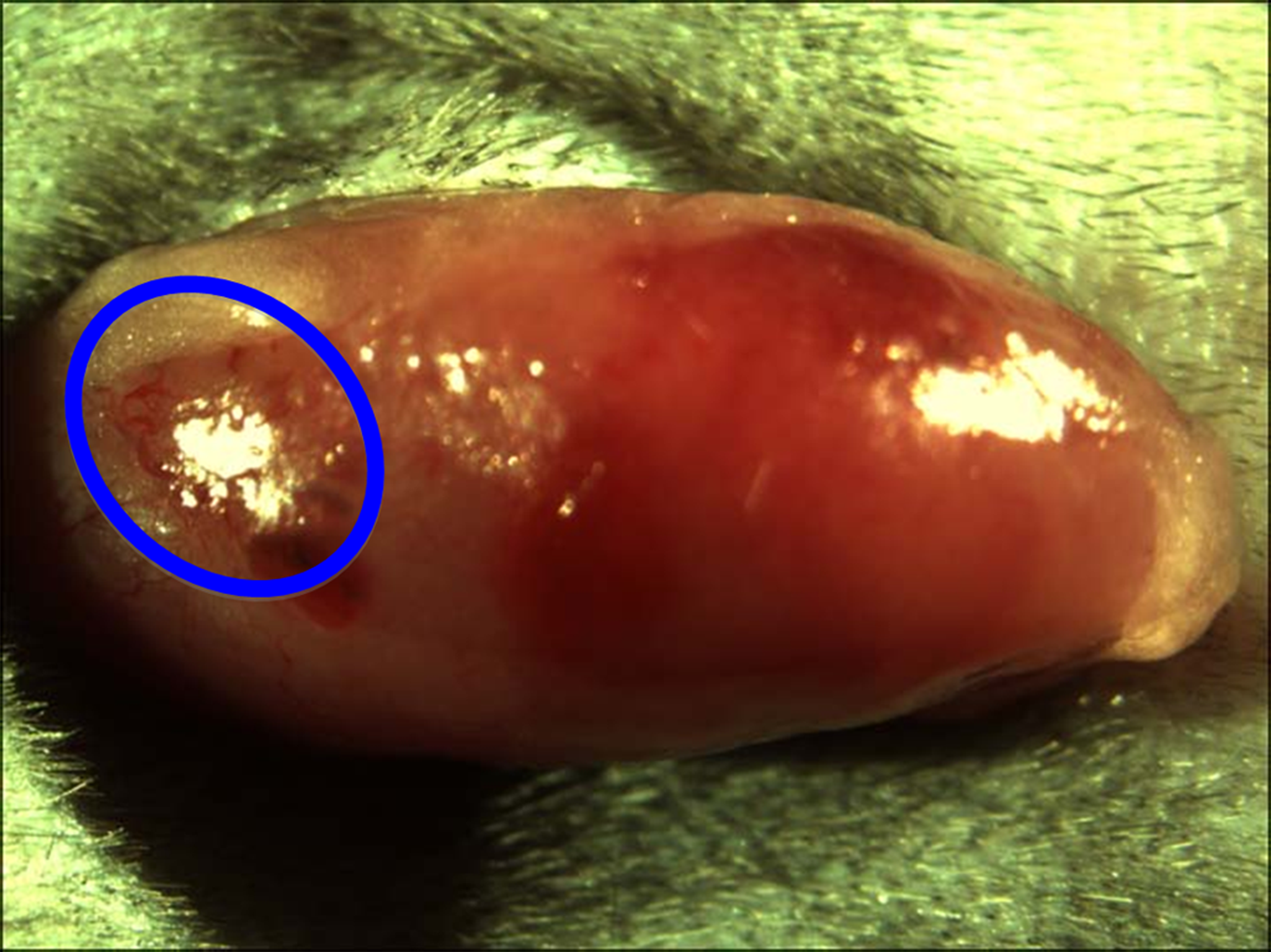

Supplement: Figure S1 — A mass of blood vessels (highlighted area) was observed near the fetal heart at one week post fetal heart transplantation to the kidney capsule. (TIF) [file pone.0031099.s002.tif]
